# Effects of an F18 enterotoxigenic *Escherichia coli* challenge on growth performance, immunological status, and gastrointestinal structure of weaned pigs and the potential protective effect of direct-fed microbial blends

**DOI:** 10.1093/jas/skaa113

**Published:** 2020-04-17

**Authors:** Spenser L Becker, Qingyun Li, Eric R Burrough, Danielle Kenne, Orhan Sahin, Stacie A Gould, John F Patience

**Affiliations:** 1 Department of Animal Science, Iowa State University, Ames, IA; 2 Department of Veterinary Diagnostic and Production Animal Medicine, Iowa State University, Ames, IA

**Keywords:** direct-fed microbials, *E. coli*, immune response, intestinal barrier, probiotics, swine

## Abstract

The objective of this experiment was to investigate the impact of an F18 enterotoxigenic *Escherichia coli* (**ETEC**) challenge on growth performance, aspects of intestinal function, and selected immune responses of piglets, as well as to evaluate potential protective effects of direct-fed microbial (**DFM**) blends. Seventy-two weaned piglets (6.4 ± 0.2 kg body weight [**BW**]; ~21 d of age) were assigned to one of four treatments: 1) **NC**: Nonchallenged (*n* = 10), 2) positive challenged control (**PC**): F18 ETEC-challenged (*n* = 10), 3) PC + **DFM1** (*n* = 8; three strains of *Bacillus amyloliquefaciens*; 7.5 × 10^5^ colony-forming units [**cfu**]/g), or 4) PC + **DFM2** (n=8; 2 strains of *B. amyloliquefaciens* and one strain of *Bacillus subtilis*; 1.5 × 10^5^ cfu/g). Feed intake and BW were recorded on day 0, 7, and 17. Pigs were sham-infected either with 6 mL phosphate-buffered saline or inoculated with 6 mL F18 ETEC (~1.9 × 10^9^ cfu/mL) on day 7 (0 d postinoculation [**dpi**]). All ETEC-challenged pigs were confirmed to be genetically susceptible to F18. Pigs had ad libitum access to feed and water throughout the 17-d trial. Fecal scores were visually ranked and rectal temperatures were recorded daily. To evaluate ETEC shedding, fecal swabs were collected on dpi 0, 1, 2, 3, 5, 7, and 10. Blood samples were collected on dpi 0, 1, 2, 4, 7, and 10. Ileal tissues were collected at necropsy on dpi 10. All challenged treatments had lower final BW, decreased average daily gain (**ADG**), and average daily feed intake (**ADFI**) during the 10-d postchallenge period (*P* < 0.01). The DFM2 treatment increased *E. coli* shedding on dpi 2 and decreased iton dpi 7 (*P* < 0.05) compared with the PC. Rectal temperature decreased across all challenged treatments (*P* < 0.01). Ileal mRNA abundance of *occludin* (***OCLN***) and *zonula occludens*-1 (***ZO-1***) decreased in PC and DFM1 compared with NC (*P* < 0.05). Pigs fed DFM2 had intermediate ileal mRNA abundance of *OCLN* and increased *ZO-1* mRNA compared with pigs in PC (*P* < 0.05). Interleukin 8 (**IL-8**) increased in the plasma of PC and DFM2 on dpi 2 compared with NC (*P* < 0.05). Mucosal IL-8 increased in PC compared with NC (*P* < 0.05). All challenged treatments tended to have elevated tumor necrosis factor-α (**TNF-α**) mRNA abundance compared with NC (*P* < 0.10). Challenged pigs had reduced secretory immunoglobulin A and villus height compared with NC pigs (*P* < 0.05). The impact of an ETEC challenge on intestinal function and the immune system has been revealed, information critical to developing improved treatment regimes.

## Introduction

There is a growing interest in reducing or eliminating antibiotic use in livestock production due to growing regulatory constraints (FDA, 2018), concerns about antimicrobial resistance (Tang et al., 2017), and consumer pressure. Antibiotic removal from weaned pig diets has substantial consequences compared to other production stages due to social and environmental stressors adversely affecting gastrointestinal function and the immune system, leading to increased incidence of disease, including diarrhea (Hao et al., 2014). Enterotoxigenic *Escherichia coli* (**ETEC**) infections are a major cause of postweaning diarrhea (**PWD**) in nursery piglets worldwide (Nagy and Fekete, 2005). Attachment of ETEC to the intestinal epithelium occurs via fimbria, commonly F4 or F18 (Zhang et al., 2007). This leads to subsequent pathogen proliferation and secretion of enterotoxins, resulting in secretory diarrhea in nursery pigs. A decrease in the rate and efficiency of body weight gain combined with increased death loss following an ETEC infection results in considerable economic losses. Vaccines and antibiotics have been used to prevent and control ETEC infections in nursery barns for many years; however, alternative strategies are needed. Direct-fed microbials (**DFM**) have been utilized in both human nutrition and livestock production for many years and are being investigated as a means of preventing and mitigating PWD in piglets. DFM are defined as products that “contain live naturally occurring microorganisms” Veterinary Medicine, 2015. These products typically consist of single or multiple bacterial strains and can include *Bacillus* species. In pigs, DFMs have been shown to enhance growth performance (Alexopoulus et al., 2004), improve intestinal function (Scharek et al., 2007; Yang et al., 2016), and positively modulate immune responses and microbial populations (Lan et al., 2016). Despite these observed beneficial effects, results derived from swine studies are inconsistent. Currently, there is limited information regarding the impact of multistrain, *Bacillus*-based DFM supplementation in F18 ETEC-challenged pigs. Therefore, the objective of this study was to characterize the impact of an F18 ETEC challenge on growth performance, aspects of intestinal barrier function, and selected immune response of weaned pigs while concurrently determining if DFMs provide protection to infected pigs. It was hypothesized that the DFMs would enhance growth performance and thus provide some degree of protection against an ETEC-challenge.

## Materials and Methods

All experimental procedures adhered to guidelines for the ethical and humane use of animals for research according to the Guide for the Care and Use of Agricultural Animals in Research and Teaching (FASS, 2010) and were approved by the Iowa State University Institutional Animal Care and Use Committee (IACUC #8-17-8576-S).

### Animals, diets, and experimental design

A total of 72 weaned pigs (6.4 ± 0.2 kg body weight [**BW**]; ~21 d of age; L337 × Camborough, PIC, Hendersonville, TN) were individually weighed and allotted to pens such that there was one barrow and one gilt per pen. Pens were randomly assigned to one of four treatments: a nonchallenged control (**NC**; *n* = 10), an ETEC-challenged control (positive challenged control [**PC**]; *n* = 10), PC + DFM1 (three strains of *Bacillus amyloliquefaciens;***DFM1**; *n* = 8), and PC + DFM2 (two strains of *B. amyloliquefaciens* and one strain of *Bacillus subtilis*; **DFM2**; *n* = 8). The DFM1 and DFM2 were included at 0.03% of the diet to achieve a final dose of 7.5 × 10^5^ cfu/g of feed and 1.5 × 10^5^ cfu/g of feed, respectively (Danisco Animal Nutrition, Marlborough, UK). The control diet was presented in mash form and was primarily based on corn and soybean meal with 9.0% whey powder and 8.5% enzymatically treated soybean meal. The DFM1 and DFM2 were added at the expense of corn in the control diet formulation. The diets were formulated to meet or exceed NRC (2012) nutrient recommendations of weaned pigs and did not contain antibiotics or pharmaceutical levels of copper or zinc (Table 1).

**Table 1.** Ingredient and nutrient composition of the experimental diets (as-fed basis, %)

Item	Control	Control+DFM1^1^	Control+DFM2^2^
Ingredient, %			
Corn	58.07	58.04	58.04
Soybean meal	15.00	15.00	15.00
Whey powder	9.00	9.00	9.00
HP300^3^	8.50	8.50	8.50
Fishmeal	4.00	4.00	4.00
Soybean oil	2.00	2.00	2.00
Limestone	1.27	1.27	1.27
Monocalcium phosphate	0.10	0.10	0.10
Salt	0.68	0.67	0.67
Vitamin premix^4^	0.20	0.20	0.20
Trace mineral premix^5^	0.20	0.20	0.20
L-Lysine HCl	0.52	0.52	0.52
DL-Methionine	0.19	0.19	0.19
L-Threonine	0.16	0.16	0.16
L-Valine	0.06	0.06	0.06
L-Tryptophan	0.03	0.03	0.03
Phytase^6^	0.02	0.02	0.02
DFM1	--	0.03	--
DFM2	--	--	0.03
Calculated nutrient levels			
ME, kcal/kg	3,407	3,407	3,407
NE, kcal/kg	2,559	2,559	2,559
Crude protein, %	20.34	20.34	20.34
Ether extract, %	4.92	4.92	4.92
Total P, %	0.53	0.53	0.53
STTD P, %	0.43	0.43	0.43
Calcium, %	0.85	0.85	0.85
SID Lys, %	1.40	1.40	1.40
SID Met + Cys, %	0.77	0.77	0.77
SID Thr, %	0.83	0.83	0.83
SID Trp, %	0.24	0.24	0.24
Analyzed nutrient levels			
Dry matter, %	85.52	85.63	85.45
GE, kcal/kg	4,329	4,338	4,321
Crude protein, %	21.51	22.32	21.68
aEE, %	5.69	5.56	5.82

^1^DFM1 Three strains of *Bacillus amyloliquefaciens;* 7.5 × 10^5^ cfu/g of feed, Danisco Animal Nutrition.

^2^DFM2 Two strains of *Bacillus amyloliquefaciens* and one strain of *Bacillus subtilis;* 1.5 × 10^5^ cfu/g of feed, Danisco Animal Nutrition.

^3^Enzymatically treated soybean meal; Hamlet Protein, Findlay, OH.

^4^Provided per kg of diet: 7,656 IU vitamin A, 875 IU vitamin D, 63 IU vitamin E, 4 mg vitamin K, 70 mg niacin, 34 mg pantothenic acid, 14 mg riboflavin, and 0.06 mg vitamin B_12._

^5^Provided per kg of diet: 165 mg Zn (zinc sulfate), 165 mg Fe (iron sulfate), 39 mg Mn (manganese sulfate), 17 mg Cu (copper sulfate), 0.3 mg I (calcium iodate), and 0.3 mg Se (sodium selenite).

^6^2,000 FTU/kg of feed provided 0.109% available P; AxtraPhy, Danisco Animal Nutrition.

This trial was conducted in a biosecurity level 2 facility at Iowa State University. Pigs were housed in one of two separate rooms based on their challenge status: One smaller room with 20 NC control pigs (10 pens) and a second larger room with 52 challenged pigs (26 pens). Room temperature, humidity, and lighting were carefully monitored throughout the trial to ensure equivalency of the room conditions. Room temperature was maintained at an average of 29.4 °C and 29.9 °C for the NC and challenged room, respectively, throughout the entire study. All pens were of equal space and flooring material. A four-space polyethylene dry feeder and one nipple drinker were used to provide ad libitum access to feed and water throughout the 17-d experiment. To avoid ETEC contamination in the nonchallenged room, strict biosecurity protocols were followed. Pigs’ genetic susceptibility to F18 ETEC was tested via Sanger DNA sequencing of the α (1,2) fucosyltransferase-1 (**FUT1**) gene according to Frydendahl et al. (2003). The F18 ETEC strain used in this study was initially isolated from the intestine of a nursery pig with colibacillosis and obtained from the culture collection at the Iowa State University Veterinary Diagnostic Laboratory (**ISU VDL**; Ames, IA). After 7 d of acclimation (0 d postinoculation [**dpi**]), pigs were orally gavaged with 6 mL of freshly grown F18 ETEC inoculum (approximately 1.9 × 10^9^ cfu/mL) or sham-infected with phosphate-buffer solution (**PBS**). The sows and piglets used in this experiment had not been previously vaccinated against *E. coli*.

### Inoculum preparation

A fluoroquinolone-resistant hemolytic *E. coli* isolate with an enrofloxacin minimal inhibitory concentration > 2 μg/mL was used to prepare the bacterial inoculum at the ISU VDL. A resistant isolate was selected in order to improve the specificity of recovery via selective media postinoculation. Briefly, a frozen culture stock of the isolate was grown (~16 h at 37 °C) on blood agar (tryptic soy agar [**TSA**] with 5% sheep blood) and was used to inoculate two bottles, each containing 50 mL of sterile tryptic soy broth. The bottles were incubated overnight at 37 °C with shaking. The broth cultures were then transferred to two new sterile bottles each with 450 mL fresh tryptic soy broth and incubated for an additional 5 h at 37 °C with shaking. The bacterial culture was centrifuged and the pellet was suspended in 900 mL of sterile PBS. The OD_600_ of the culture in PBS was measured to be 4.25 using a spectrophotometer (Bio-Rad SmartSpec 3000, Hercules, CA). A viable CFU count was performed and the inoculum was determined to have approximately 1.9 × 10^9^ cfu/mL.

### Sample collection

Pigs were individually weighed on dpi −7, 0, and 10. Feed disappearance was recorded to calculate average daily gain (**ADG)**, average daily feed intake (**ADFI**), and gain to feed ratio (**G:F**) for each phase. On dpi 0, 1, 2, 3, 5, 7, and 10, rectal fecal swabs were collected from one barrow per pen to evaluate F18 ETEC shedding. Rectal temperatures were obtained daily from every pig via rapid-response digital electric thermometers (ReliOn, MABIS Healthcare Inc., Waukegan, IL). Pen fecal score was visually assessed daily by two unbiased personnel using the following scale: 0 = solid, 1 = semi-solid, 2 = semiliquid, and 3 = liquid. Fecal score ≥ 2 was considered diarrhea. On dpi 0 (immediately before inoculation), 1, 2, 4, 7, and 10, blood samples were collected from one barrow per pen via jugular venipuncture into a 10 mL heparin vacutainer tube (Bection Dickinson, Franklin Lakes, NJ). Plasma was separated by centrifugation (2,000 × *g* for 10 min at 4 °C), divided into three aliquots, and stored at −80 °C for later analysis.

On dpi 10, one pig from each pen was euthanized by captive bolt stunning followed by exsanguination. Post-euthanasia, the abdomen was opened and a 30 cm segment of ileum anterior to the ileocecal junction was removed, drained of digesta, and rinsed with ice-cold PBS. Three 2 cm segments of the terminal ileum were fixed in 10% neutral buffered formalin. The remaining ileal segments were snap frozen in liquid N and stored at −80 °C for later analysis. A second 20 cm segment of ileum was removed, snap frozen in liquid N, and stored at −80 °C for harvesting of mucosal scrapings.

### Chemical analysis

Diets were ground to 1 mm particle size with a Wiley Mill (Variable Speed Digital ED-5 Wiley Mill; Thomas Scientific, Swedesboro, NJ) and analyzed in duplicate for DM (method 930.15 [AOAC, 2007]), acid-hydrolyzed ether extract (**aEE**; method 2003.06; [AOAC, 2007]), and N (method 990.03 [AOAC, 2007]; TruMac; LECO Corp., St. Joseph, MI). An ethylenediaminetetraacetic acid sample (9.56% N) was used as the standard for calibration and was determined to contain 9.55 ± 0.01% N. Crude protein was calculated as N × 6.25. Gross energy was determined in duplicate using an isoperibolic bomb calorimeter (model 6200; Parr Instrument Co., Moline, IL). Benzoic acid (6,318 kcal GE/kg) was used as the standard for calibration and was determined to contain 6,319 ± 0.8 kcal GE/kg.

### Fecal F18 ETEC shedding

For isolation of *E. coli* from fecal samples, fecal swabs were plated onto selective TSA agar with 5% bovine blood, 16 µg ciprofloxacin/mL, and 50 µg cycloheximide/mL and onto MacConkey agar. Plates were incubated at 37 °C for 24 h to determine hemolytic *E. coli* shedding using a semiquantification method. Shedding of ETEC was measured using a 5-point scale ranging from 0 to 4 according to the number of streaked sections that had viable *E. coli*, where 0 corresponded to no growth, 1 corresponded to growth in the primary streak, 2 corresponded to compatible growth extending into the secondary streak, 3 corresponded to growth into the tertiary streak, and 4 corresponded to growth into the quaternary section of the agar plate (Li et al., 2019). Identification of *E. coli* isolates was confirmed by matrix-assisted laser desorption ionization time-of-flight mass spectrometry (**MALDI-TOF MS**; Singhal et al., 2015) at the **ISU VDL** following the standard methods.

### Ileal *E. coli* attachment

Formalin-fixed ileum tissues were processed and embedded in paraffin wax at the ISU VDL. Three transverse sections (5 μm) were cut from the ileum, stained with hematoxylin and eosin, and mounted on glass slides. Visualization of *E. coli* attachment to epithelial cells was accomplished using an OLYMPUS BX 53/54 microscope at 40× power. Each section was scored as either 0 if there was no attachment or 1 if there was attachment of *E. coli* on ≥ 5 villi in each section. The *E. coli* attachment frequency (%) was calculated by summing up the score of all three sections on each glass slide and then dividing by 3.

### Intestinal morphology

Images of ileal sections were taken using a DP80 Olympus Camera mounted on an OLYMPUS BX 53/43 microscope with a motorized stage. Whole ileal sections were scanned at 4× power, then regions containing well-orientated villus and crypt pairs were selected. These regions were rescanned at 20× power. The 20× regions were stitched together to form a composite image. Ten well-orientated villus and crypt pairs per ileal section per slide were selected and villus height and crypt depth were measured using OLYMPUS cellSens Dimension 1.16 software.

### RNA isolation and quantitative PCR

Approximately 30 mg of ileal tissue was homogenized using the Qiagen Tissuelyser II (Germantown, MD); then total RNA was isolated using the Qiagen RNeasy Mini Kit according to the manufacturer’s recommendations. The concentration of RNA was quantified using a spectrophotometer (ND-100; NanoDrop Technologies, Inc., Rockland, DE). All samples had 260:280 nm ratios above 1.8. The QuantiTect Reverse Transcription Kit (Qiagen GmbH, Hilden, Germany) was used according to the manufacturer’s instructions to synthesize complementary DNA (**cDNA**) from 0.8 μg of the isolated RNA. All cDNA samples were diluted 10-fold with nuclease-free water.

Real-time quantitative polymerase chain reaction (**PCR**) was performed using iQ SYBR Green Supermix (Bio-Rad Laboratories, Inc., Hercules, CA). The gene-specific primers, shown in Table 2, were diluted to 10 µM with nuclease-free water. Genes were chosen to evaluate small intestinal inflammatory status and paracellular permeability. Ribosomal protein-L19 (**RPL19**) was included as an endogenous reference gene. Each reaction included 10 µL of SYBR Green Supermix, 1 µL of each forward and reverse primer, 5 µL of nuclease-free water, and 3 µL of cDNA, for a total of 20 µL reaction volume. Each 96-well plate contained a no-reverse transcriptase negative control and a pooled cDNA reference sample. Samples were assayed in triplicate. Fluorescence of SYBR Green was quantified with a Real-time PCR Detection System (iQ5; Bio-Rad Laboratories Inc.). Cycling conditions were as follows: 5-min initial denaturation at 95 °C followed by 40 PCR cycles (95 °C for 30 s, 55 °C for 30 s, and 72 °C for 30 s) and a dissociation curve to verify the amplification of a single PCR product. Optical detection was performed at 55 °C. Analyses of amplification plots were performed with an Optical System Software version 2.0 (iQ5; Bio-Rad Laboratories Inc.) and cycle threshold (**Ct**) values for each reaction was obtained. The mRNA abundance for each sample was normalized to *RPL19* and the pooled sample, and fold change was calculated using the 2^-ΔΔCT^method (Livak and Schmittgen, 2001).

**Table 2.** Primer sequences used for quantitative PCR

Gene	Primer sequence	Product size, bp	GenBank accession
*TNFα*	F: CACCACGCTCTTCTGCCTAC	132	X57321
	R: ACGGGCTTATCTGAGGTTTGAGACG		
*CLDN1*	F: GATTTACTCCTACGCTGGTGAC	199	AJ318102
	R: CACAAAGATGGCTATTAGTCCC		
*CLDN3*	F: TTGCATCCGAGACCAGTCC	85	NM_001160075
	R: AGCTGGGGAGGGTGACA		
*OCLN*	F: AACTCCCGTCAGCAGATCC	95	NM_001163647
	R: ATCAGTGGAAGTTCCTGAACCA		
*ZO-1*	F: CTCTTGGCTTGCTATTCG	197	XM_003353439
	R: AGTCTTCCCTGCTCTTGC		
*TLR4*	F: CAGATAAGCGAGGCCGTCATT	113	AB232527
	R: TTGCAGCCCACAAAAAGCA		
*CD14*	F: CCTCAGACTCCGTAATGTG	180	AB267810
	R: CCGGGATTGTCAGATAGG		
*RPL19*	F: AACTCCCGTCAGCAGATCC	147	AF435591
	R: AGTACCCTTCCGCTTACCG		

### Mucosal disaccharidase activity, secretory IgA, and cytokines

Ileal mucosal scrapings (0.5 g) were added to 4.5 mL of PBS containing a protease inhibitor cocktail (Sigma Aldrich, St. Louis, MO) and triton (0.1%). The resulting solution was homogenized and centrifuged at 10,000 × *g* for 15 min at 4 °C and the supernatant was stored in aliquots. Total protein concentration of hydrolyzed mucosa was quantified using a Pierce bicinchoninic acid Protein Assay kit (Thermo Scientific, Woltham, MA). The intra-assay coefficient of variation was 6.4%. Disaccharidase-specific activity was determined as previously described by Dahlqvist (1964) using lactose, maltose, and sucrose as substrates. The intra- and interassay coefficients of variation was 7.3 and 14.7%. Enzyme activity was expressed as μmol hydrolyzed substrate × min-1×g tissue protein-1. Concentration of secretory IgA was obtained using a porcine-specific ELISA kit following manufacturer’s instructions (Bethyl Laboratories, Inc., Montegomery, TX). The intra-assay coefficient of variation was 3.8%. Homogenized mucosa and plasma subsamples were analyzed for cytokines using a Multiplex Immunoassay (Eve Technologies, Calgary, AB, Canada).

### Statistical analysis

Data were analyzed as a complete randomized design. Pen was the experimental unit and treatment was a fixed effect. Growth performance data and plasma cytokines were analyzed as repeated measures with pen as the experimental unit using the MIXED procedure of SAS (SAS Institute Inc., Cary, NC) with a spatial power covariance structure. Baseline (day 0) measurements of plasma cytokines were used as a covariate. ETEC shedding scores were analyzed using PROC MIXED as repeated measures using a spatial power covariance structure with pig as a random effect. Averaged fecal scores and rectal temperatures were analyzed using PROC MIXED as repeated measures with a first order autoregressive covariance structure. Mucosal cytokines, morphology, secretory IgA, disaccharidase, and mRNA abundance data were analyzed in PROC MIXED. The ETEC attachment data were analyzed in PROC GLIMMIX assuming a binomial distribution. Least square means of treatments were reported. Preplanned contrasts were performed using the ESTIMATE statement to evaluate the effects of the ETEC challenge (NC vs. PC) and dietary treatment (PC vs. DFM1, DFM2). For each variable, normal distribution of residuals was tested using PROC UNIVARIATE. Differences were considered significant if *P* was ≤ 0.05 and a tendency if *P* was > 0.05 and ≤ 0.10.

## Results

### Growth performance and mortality

No mortality was observed prior to the challenge. Following ETEC challenge, 23% mortality was observed among the challenge pigs. There were no differences among treatments (data not shown). In all instances, it was confirmed via necropsy that mortality was due to severe dehydration. During the 7-d adaptation period, there were no significant differences in ADG or ADFI among the four treatments (Table 3; *P* ≥ 0.466). Pigs receiving either DFM product had increased G:F during the 7-d adaptation period (*P* = 0.005) compared to the control pigs. Pig BW did not differ on dpi −7 or dpi 0 (*P* ≥ 0.785). The pigs within the PC, DFM1, and DFM2 treatments had lower final BW (*P* <0.001) and lower ADG (*P* < 0.001) during the 10-d challenge period compared with the NC. The NC pigs also had a higher feed intake than pigs on the other three treatments (*P* < 0.001). The G:F during postchallenge period was not different among all treatments (*P* = 0.203).

**Table 3.** Effects of treatment on growth performance in weaned pigs challenged with F18 ETEC

	Treatment^1^					
Item	NC	PC	DFM1	DFM2	SEM	*P*-value
BW, kg						
dpi −7	6.59	6.59	6.34	6.17	0.17	0.785
dpi 0	6.88	6.83	6.73	6.80	0.17	0.990
dpi 10	10.78^a^	9.22^b^	8.55^b^	9.17^b^	0.17	<0.001
dpi −7 to 0						
ADG, kg	0.04	0.03	0.05	0.09	0.01	0.466
ADFI, kg	0.08	0.09	0.08	0.11	0.01	0.859
G:F^2^	0.45^b^	0.34^b^	0.67^ab^	0.79^a^	0.06	0.035
dpi 1 to 10						
ADG, kg	0.39^a^	0.19^b^	0.15^b^	0.22^b^	0.01	<0.001
ADFI, kg	0.49^a^	0.33^b^	0.19^b^	0.33^b^	0.01	<0.001
G:F	0.81	0.54	0.51	0.65	0.06	0.203

^1^NC (*n* = 10); PC (*n* = 9); DFM 1 = PC + direct-fed microbial 1 (*n* = 8; three strains of *Bacillus amyloliquefaciens;* 7.5 × 10^5^ cfu/g of feed); DFM 2 = PC + direct-fed microbial 2 (*n* = 7; two strains of *B. amyloliquefaciens* and one strain of *Bacillus subtilis;* 1.5 × 10^5^ cfu/g of feed). Supplementation rates were based on manufacturer’s recommendations (Danisco Animal Nutrition).

^2^Interpretation of G:F should be cautious because values less than −1.4 were removed from analysis (2 numbers prechallenge from PC and DFM1). Additionally, three pigs in PC had G:F ranging from −0.47 to −0.07 and one pig from DFM1 had a G:F = −0.56. Five pigs with G:F > 1 from both DFM treatments during dpi −7 to 0 were included in the analysis.

^a,b^Means with differing superscripts indicate a significant (*P* < 0.05) difference.

### ETEC shedding, fecal score, and rectal temperature

**Figure 1.**
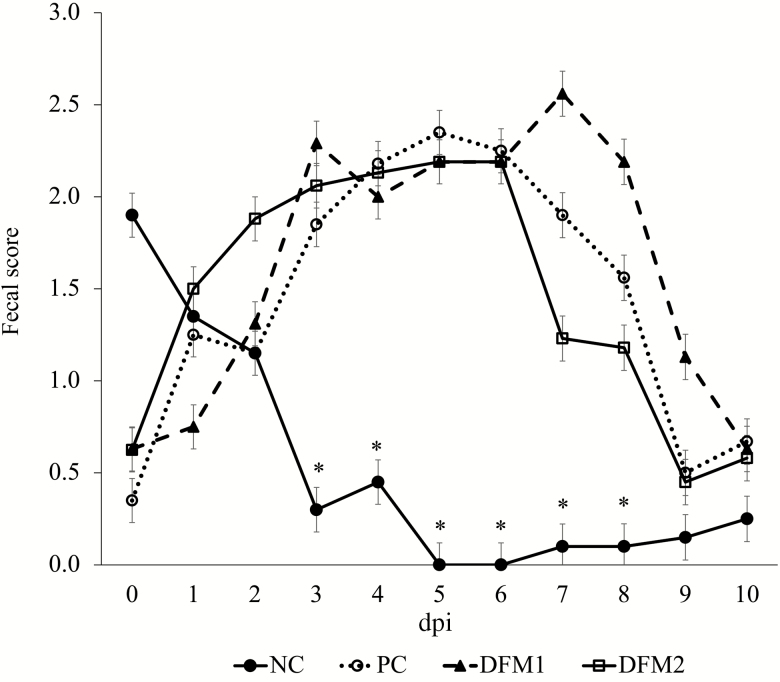
Effects of treatment on the daily fecal score of pigs challenged with F18 ETEC. NC (*n* = 10); PC (*n* = 9); DFM1 = PC + direct-fed microbial 1 (*n* = 8; three strains of *Bacillus amyloliquefaciens;* 7.5 × 10^5^ cfu/g of feed); DFM2 = PC + direct-fed microbial 2 (*n* = 7; two strains of *B. amyloliquefaciens* and one strain of *Bacillus subtilis;* 1.5 × 10^5^ cfu/g of feed). Supplementation rates were based on manufacturer’s recommendations (Danisco Animal Nutrition). *P* (NC vs. PC; day postinoculation (dpi) 3) < 0.001, *P* (PC vs. DFM1, DFM2; dpi 3) > 0.10, *P* (all treatments; dpi 10) > 0.10.

**Figure 2.**
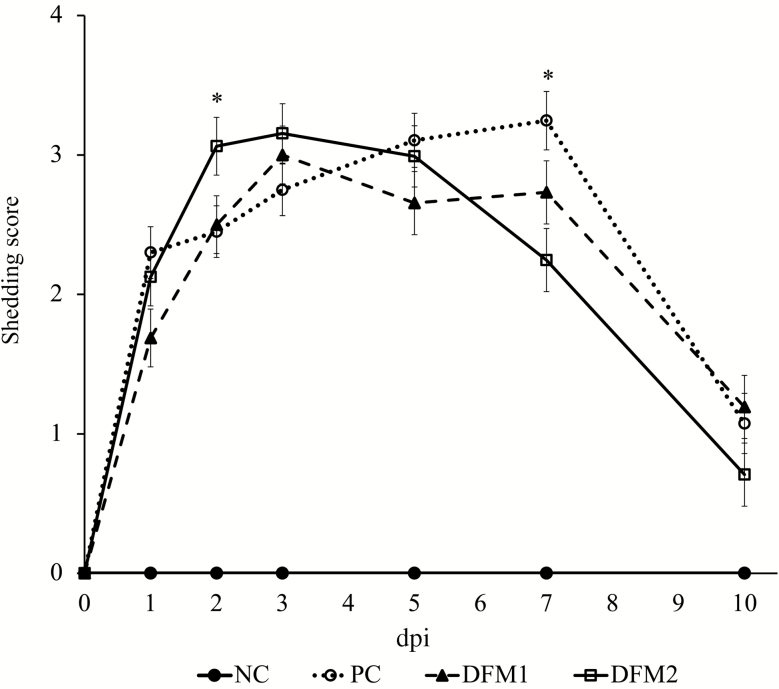
Effects of treatment on *E. coli* shedding score of pigs challenged with F18 ETEC. NC (*n* = 10); PC (*n* = 9); DFM1 = PC + direct-fed microbial 1 (*n* = 8; three strains of *Bacillus amyloliquefaciens;* 7.5 × 10^5^ cfu/g of feed); DFM2 = PC + direct-fed microbial 2 (*n* = 7; two strains of *B. amyloliquefaciens* and one strain of *Bacillus subtilis;* 1.5 × 10^5^ cfu/g of feed). Supplementation rates were based on manufacturer’s recommendations (Danisco Animal Nutrition). *P* (NC vs. PC; overall period) < 0.001, *P* (PC vs. DFM1; overall period) > 0.10, *P* (PC vs. DFM2; dpi 2) = 0.044, *P* (PC vs. DFM2; dpi 7) = 0.003, *P* (all treatments; dpi 10) > 0.10.

**Figure 3.**
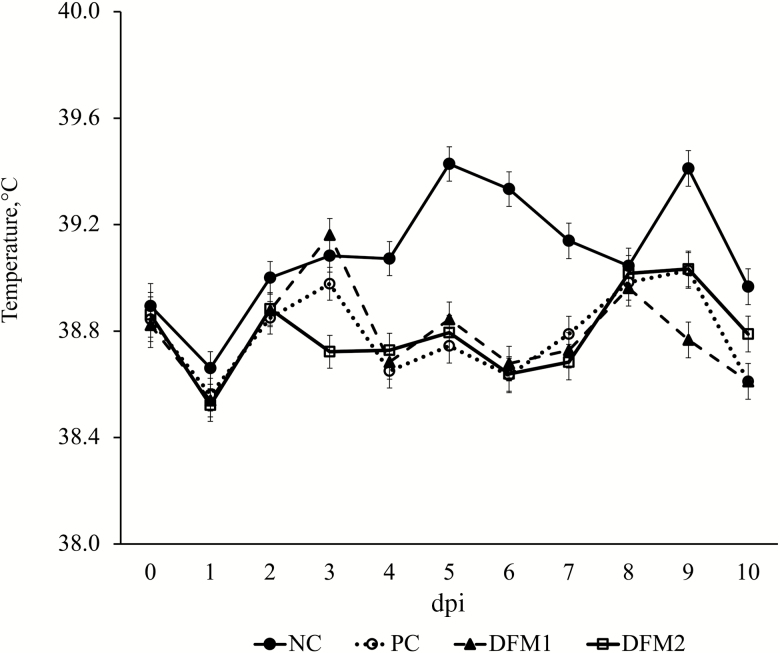
Effects of treatment on daily rectal temperature of pigs challenged with F18 ETEC. NC (*n* = 10); PC (*n* = 9); DFM1 = PC + direct-fed microbial 1 (*n* = 8; three strains of *Bacillus amyloliquefaciens;* 7.5 × 10^5^ cfu/g of feed); DFM2 = PC + direct-fed microbial 2 (*n* = 7; two strains of *B. amyloliquefaciens* and 1 strain of *Bacillus subtilis;* 1.5 × 10^5^ cfu/g of feed). Supplementation rates were based on manufacturer’s recommendations (Danisco Animal Nutrition). *P* (NC vs. PC; overall period) < 0.0001, *P* (PC vs. DFM1; overall period) = 0.962; *P* (PC vs. DFM2; overall period) = 0.947.

Overall fecal score for the 10-d challenge period for NC was lower compared with the challenged treatments (*P* < 0.001, Figure 1). There were no differences in fecal scores among challenged treatments over the 10-d challenge period (*P* > 0.10). Prior to ETEC challenge, all pigs were confirmed negative for ETEC shedding. Pigs on NC had no ETEC shedding throughout the experiment (Figure 2). The PC pigs had higher shedding over the 10-d challenge period compared with NC (*P* < 0.05). The DFM2 treatment increased ETEC shedding score (SS) on dpi 2 (*P* = 0.044) and decreased SS on dpi 7 (*P* = 0.003) compared with PC. There were no differences in SS between PC and DFM1 (*P* > 0.10). On dpi 10, there were no differences in SS among all treatments (*P* > 0.10). For the overall 10-d challenge period, there was a significant reduction in rectal temperature across all challenged pigs compared with NC (*P* < 0.001; Figure 3). There were no differences in rectal temperature among challenged pigs (*P* > 0.10).

### Mucosal and plasma cytokines

**Figure 4.**
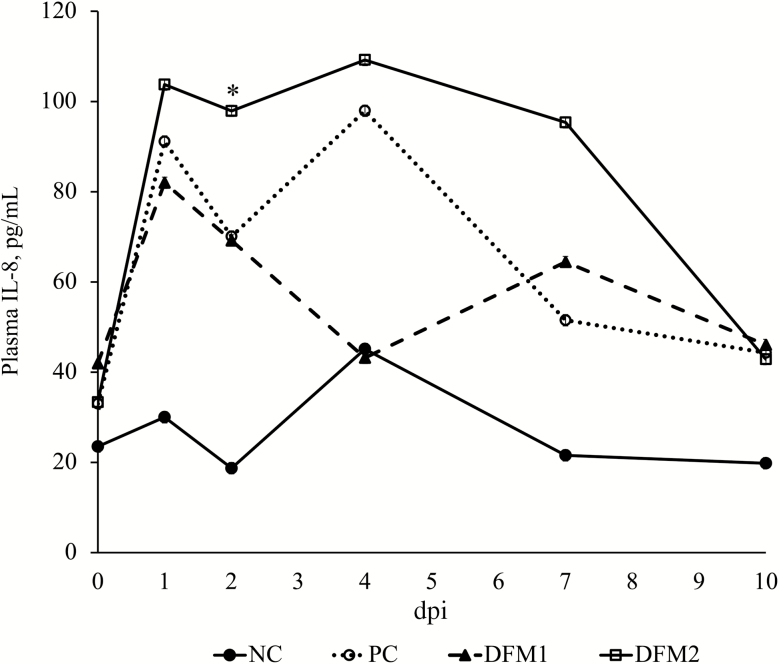
Effects of treatment on plasma IL-8 of pigs challenged with F18 ETEC. NC (*n* = 10); PC (*n* = 9); DFM1 = PC + direct-fed microbial 1 (*n* = 8; three strains of *Bacillus amyloliquefaciens*; 7.5 × 10^5^ cfu/g of feed); DFM2 = PC + direct-fed microbial 2 (*n* = 7; two strains of *B. amyloliquefaciens* and one strain of *Bacillus subtilis;* 1.5 × 10^5^ cfu/g of feed). Supplementation rates were based on manufacturer’s recommendations (Danisco Animal Nutrition). *P* (NC vs. PC; dpi 1) = 0.069; *P* (NC vs. PC; dpi 2) = 0.031; *P* (PC vs. DFM1, DFM2; overall period) > 0.10; *P* (all treatments; dpi 4, 7, 10) > 0.10.

Analysis of ileal mucosal cytokines revealed no differences among treatments for interferon-γ (**IFNγ**), interleukin-1rα (**IL-1rα**), IL-1α, IL-1β, IL-2, IL-4, IL-6, IL-10, IL-12, or IL-18 (*P* > 0.10; Table 4). Mucosal IL-8 was elevated in PC compared with NC (*P* = 0.011); however, there were no differences among challenged treatments. Similarly, there were no differences in plasma cytokines among treatments (data not shown) with the exception of IL-8. While there were no differences among challenged treatments, IL-8 tended to be elevated in PC vs. NC (*P* = 0.069; Figure 4) on dpi 1 and significantly elevated on dpi 2 (*P* = 0.031). There were no differences in plasma IL-8 on dpi 4, 7, or 10 among all treatments (*P* > 0.10).

**Table 4.** Effect of treatment on mucosal cytokines^2^, disaccharidase activity^3^, and secretory immunoglobulin A^4^ in the ilea of weaned pigs challenged with F18 ETEC

	Treatment^1^					
Item	NC	PC	DFM1	DFM2	SEM	*P*-value
IFNγ	7.04	7.96	5.07	7.47	1.57	0.521
IL-1rα	20.56	24.06	19.21	16.48	3.69	0.828
IL-1α	1.66	1.88	2.11	1.70	0.27	0.421
IL-1β	8.30	9.59	12.08	7.55	1.59	0.397
IL-2	0.49	0.69	0.57	0.52	0.11	0.465
IL-4	0.79	0.48	0.58	0.48	0.17	0.508
IL-6	0.35	0.57	0.86	0.24	0.20	0.212
IL-8	240.20^b^	343.06^a^	288.07^ab^	283.42^ab^	29.13	0.011
IL-10	0.30	0.25	0.27	0.25	0.04	0.302
IL-12	3.06	3.81	3.14	4.35	0.44	0.214
IL-18	220.69	227.45	223.82	229.00	27.64	0.854
Lactase	0.07	0.11	0.03	0.13	0.05	0.511
Sucrase	1.81^c^	4.49^a^	2.56^b^	4.72^a^	0.58	0.003
Maltase	7.39^c^	14.16^ab^	9.61^bc^	20.91^a^	2.64	0.004
sIgA	2.50^a^	1.11^b^	1.37^b^	0.95^b^	0.33	0.011

^1^NC (*n* = 10); PC (*n* = 9); DFM1 = PC + direct-fed microbial 1 (*n* = 8; three strains of *Bacillus amyloliquefaciens;* 7.5 × 10^5^ cfu/g of feed); DFM2 = PC + direct-fed microbial 2 (*n* = 7; two strains of *B. amyloliquefaciens* and 1 strain of *Bacillus subtilis;* 1.5 × 10^5^ cfu/g of feed). Supplementation rates were based on manufacturer’s recommendations (Danisco Animal Nutrition).

^2^ng/g of mucosa.

^3^U/min/g of protein.

^4^μg/mg of protein.

^a–c^ Means with differing superscripts indicate a significant (*P* < 0.05) difference.

### Mucosal secretory IgA and disaccharidase activities

Secretory IgA was reduced in ileal mucosa of all challenged treatments compared with the NC (*P* = 0.011; Table 4). Lactase activity did not differ among treatments (*P* = 0.511). Sucrase activity increased in PC and DFM2 compared with NC (*P* = 0.003). Pigs fed DFM1 had reduced sucrase activity compared with PC. The PC and DFM2 also had increase maltase activity compared with NC (*P* = 0.004); there was no difference between PC and DFM1 (*P* > 0.10).

### Ileal gene transcription

There were no differences among treatments in toll-like receptor 4 (**TLR4**) mRNA abundance or the tight junction proteins claudin-1 (**CLDN1**) or CLDN3 (*P* > 0.10; Table 5). A trend for greater TNFα mRNA abundance was observed across all challenged treatments compared with NC (*P =* 0.087). *Occludin* (***OCLN***) mRNA abundance was significantly reduced in PC and DFM1 compared with NC (*P =* 0.045). Pigs receiving DFM2 had OCLN mRNA abundance intermediate of NC and PC. Lower *zonula-occludens-1* (***ZO-1***) mRNA abundance was observed in PC and DFM1 compared with NC and DFM2 (*P =* 0.001). Cluster of differentiation (**CD14**) was elevated in DFM2 compared with NC and PC (*P =* 0.019). Pigs receiving DFM1 had CD14 mRNA abundance intermediate of PC and DFM2.

**Table 5.** Effect of treatment on ileal gene mRNA abundance in weaned pigs challenged with F18 ETEC

	Treatment^1^					
Gene	NC	PC	DFM1	DFM2	SEM	*P*-value
*CLDN1*	1.15	0.59	0.75	1.12	0.21	0.145
*CLDN3*	0.98	0.64	0.65	0.95	0.34	0.332
*OCLN*	1.15^a^	0.48^b^	0.54^b^	0.69^ab^	0.38	0.045
*ZO-1*	1.05^a^	0.56^b^	0.67^b^	1.05^a^	0.10	0.001
*TNFα*	1.01	1.84	1.56	2.30	0.37	0.087
*CD14*	1.09^b^	1.52^b^	1.89^ab^	2.60^a^	0.34	0.019
*TLR4*	0.88	0.59	0.68	0.95	0.14	0.145

^1^NC (*n* = 10); PC (*n* = 9); DFM1 = PC + direct-fed microbial 1 (*n* = 8; three strains of *Bacillus amyloliquefaciens;* 7.5 × 10^5^ cfu/g of feed); DFM2 = PC + direct-fed microbial 2 (*n* = 7; two strains of *B. amyloliquefaciens* and one strain of *Bacillus subtilis;* 1.5 × 10^5^ cfu/g of feed). Supplementation rates were based on manufacturer’s recommendations (Danisco Animal Nutrition).

^a,b^Means with differing superscripts indicate a significant (*P* < 0.05) difference.

### Morphology and *E. coli* attachment to epithelial cells

Villus height in the ileum was reduced across all challenged treatments compared with NC (*P* < 0.001; Table 6). Ileal crypts tended to be shallower in pigs fed DFM1 (*P* = 0.074). Villus height:crypt depth was reduced in DFM1 vs. NC (*P* = 0.046). There were no differences in villus height:crypt depth among NC, PC, and DFM2. Attachment of *E. coli* to the epithelial cells in the ileum did not differ across all treatments (*P* = 0.101).

**Table 6.** Effect of treatment on ileal morphology and *E. coli* attachment in weaned pigs challenged with F18 ETEC

	Treatment^1^					
Item	NC	PC	DFM1	DFM2	SEM	*P*-value
Villus height, µm	344.41^a^	253.82^b^	206.92^c^	256.05^b^	14.56	<.001
Crypt depth µm	199.43	177.95	160.44	172.83	10.53	0.074
VH:CD^2^	1.74^a^	1.48^ab^	1.29^b^	1.54^ab^	0.11	0.046
Attachment, %	0.00	14.80	33.33	0.00	5.72	0.102

^1^NC (*n* = 10); PC (*n* = 9); DFM1 = PC + direct-fed microbial 1 (*n* = 8; three strains of *Bacillus amyloliquefaciens;* 7.5 × 10^5^ cfu/g of feed); DFM2 = PC + direct-fed microbial 2 (*n* = 7; two strains of *B. amyloliquefaciens* and one strain of *Bacillus subtilis;* 1.5 × 10^5^ cfu/g of feed). Supplementation rates were based on manufacturer’s recommendations (Danisco Animal Nutrition).

^2^Villus height:crypt depth ratio.

^a-c^Means with differing superscripts indicate a significant (*P* < 0.05) difference.

## Discussion

Rate and efficiency of body weight gain in newly weaned pigs is closely associated with intestinal health and function. Enterotoxigenic *E. coli* infections after weaning reduce feed intake and negatively impact gut health and intestinal function in the pig (Liu et al., 2013; Pan et al., 2017; Li et al., 2019). Limited research exists evaluating the impact of *Bacillus-*based DFM products in ETEC-challenged pigs. This study evaluated the effects of two novel *Bacillus-*based DFMs on growth performance, intestinal function, and immune response in weaned pigs challenged with F18 ETEC. Following inoculation with ETEC, all challenged pigs had increased fecal scores and shedding of the F18 ETEC strain compared with NC, confirming the challenge model was successful. Final BW, and postchallenge ADG and ADFI were reduced in ETEC-challenged pigs, though this did not result in a difference in feed efficiency compared with the NC. The lack of difference in feed efficiency was likely due to the significant decreases in both ADG and ADFI. The lack of difference in feed efficiency and reductions in pig growth performance due to an ETEC challenge are in agreement with previous research (Pan et al., 2017; Li et al., 2019).

While there were no differences in F18 ETEC shedding of challenged pigs across the 10-d challenge period, pigs supplemented with DFM2 had increased ETEC shedding on dpi 2, followed by a decrease in shedding on dpi 7 compared with PC. Despite the fact that the exact modes of action of *Bacillus-*based DFMs are unknown, the decrease in *E. coli* shedding on dpi 7 is possibly due to the ability of DFM2 to more rapidly utilize carbon and energy sources and successfully compete for nutrient absorption sites, thereby suppressing growth of pathogenic bacteria (Cho et al., 2011). Pathogenic bacteria, such as ETEC, must attach to the intestinal epithelial wall in order colonize and express symptoms of disease (Walker, 2000). DFM are proposed to increase the colonization of normal microbiota, thus inhibiting the adhesion of pathogenic bacteria to the intestinal epithelium (Yirga, 2015). Members of the *Bacillus* genus have also been widely reported to produce bacteriocins, which are proteins with antimicrobial properties that can inhibit activity of pathogenic bacteria (Abriouel et al., 2011; Larsen et al., 2014).

The ETEC challenge also reduced rectal temperatures across all challenged treatments compared with NC. This lack of febrile response is consistent with the absence of systemic cytokine and chemokine production, which are known to initiate a febrile response (Evans et al., 2015). Reductions in rectal temperature have been reported due to decreased feed intake (Pearce et al., 2013), which in turn presumably reduces the heat increment of feeding. Spitzer et al. (2014) also reported decreases in rectal temperature in weaned pigs following an ETEC challenge. Furthermore, it has been shown that in severe cases of sepsis, inducing fever is not favorable due to the high energy demand (Romanovsky and Székely, 1998). Rather, the body will induce hypothermia in order to conserve energy, reduce the need of oxygen, and protect vital organs (Luscombe and Andrzejowski, 2006; Schieber and Ayres, 2016).

In addition to clinical symptoms, ETEC is known to increase intestinal permeability through alterations in tight junction proteins. The reductions of *OCLN* and *ZO-1* mRNA abundance due to ETEC infection observed in this study are in agreement with other reports (Ewaschuk et al., 2011; Gao et al., 2013; Li et al., 2019). The greater mRNA abundance of *OCLN* and *ZO-1* in ETEC-challenged pigs fed DFM2 compared with PC indicates an improvement in intestinal barrier integrity, potentially resulting from the initial increases and subsequent decreases in *E. coli* shedding. The ability of *Bacillus-*based products to improve tight junction protein expression has been previously described (Gu et al., 2014; Rhayat et al., 2019). Intestinal barrier preservation is partly dependent upon tight junction proteins, as they have an important role in preventing paracellular transport of harmful bacteria and toxins across the intestine (Mukiza et al., 2013). Transport of luminal contents and pathogenic material into the peripheral circulation can increase when tight junctions are disrupted, thus activating an immune response and intestinal inflammation. Activation of intestinal inflammation was indicated by elevated levels of ileal *CD14*, a coreceptor of the TLR-4 complex, which recognizes the lipopolysaccharide component of Gram-negative bacteria (Guo et al., 2013). The upregulation of *CD14* possibly explains the increase in the proinflammatory cytokine IL-8 observed in both the intestinal mucosa and plasma of PC compared with NC, which has been previously reported following an ETEC challenge (Li et al., 2019). However, pigs receiving either DFM product had intermediate IL-8 levels in the ileal mucosa, suggesting the ability of the DFMs to blunt this response. Roselli et al. (2007) reported increased levels of IL-8 accompanied by disruption in the tight junction complex following an ETEC infection, which agrees with these results. These data provide insight into the role of IL-8 during an ETEC challenge, as it was localized in the intestine; however, there was no difference in systemic IL-8 at dpi 10. This localized proinflammatory response may be associated with the negative impacts on the intestinal barrier observed in PC. The lack of differences observed in other proinflammatory cytokines, such as IL-6 and TNFα, in both the blood and tissue are likely due to the fact that pigs were recovering from ETEC infection by necropsy day on dpi 10, which is supported by the decreases in fecal scores, ETEC shedding, and *E. coli* attachment to the intestinal epithelium.

To further evaluate intestinal immune response, sIgA was measured in the ileal mucosa. The mucosal immune system is critical in protecting the host from pathogens. Secretory IgA is the most abundant antibody found in the intestine, serving as the first line of barrier defense of the mucosal immune system in the event of an enteric infection (Mantis et al., 2011). In the current study, sIgA levels were reduced due to ETEC challenge, which is inconsistent with previous literature (Zhang et al., 2013). This is possibly explained by a mechanism used by sIgA known as immune exclusion, which inhibits the ability of pathogens and toxins to interact with the intestinal epithelium. Once bound, sIgA facilitates bacterial clearance by increasing peristaltic movement in the GIT (Mantis et al., 2011). Thus, the majority of sIgA produced may have been shed with the GIT contents. Additionally, sIgA can intercept incoming pathogens intracellularly as it is crossing the epithelial barrier, as well as neutralize pathogens that have successfully crossed the intestinal barrier into the lamina propria (Corthésy, 2013). Due to the observed impairment in intestinal barrier integrity, sIgA may be acting at these sites to neutralize pathogenic agents instead of at the mucosal surface. Polymeric IgA secreted by plasma cells in the lamina propria is able to neutralize pathogens by recognizing and binding to epitopes on the pathogen, thus rendering it unable to elicit symptoms of disease. This IgA:antigen complex is then exported across the intestinal wall via transcytosis and flushed out of the intestinal lumen (Corthésy, 2013). Additionally, it has been established that mucosal atrophy occurs with adverse morphological changes (Shaw et al., 2012). In the present study, villus height was significantly reduced across all challenged treatments, which has been associated with weaning and ETEC challenges (Pluske et al., 1997; Yang et al., 2014). The observed villus atrophy may have resulted in less mucosal mass in the intestine and therefore less surface area for sIgA to bind to.

Intestinal function was also assessed by measuring disaccharidase activity in the ileum. Intestinal disaccharidases are essential for digestion of carbohydrates. While no difference in lactase activity was observed, PC and DFM2 had increased sucrase and maltase activity compared with NC. It has been shown that DFM supplementation in pig diets increases amylase, sucrase, maltase, and Na^+^/K^+^-ATPase activities (Hu et al., 2018), which is reflective of improved intestinal function. There is little information regarding the impact of an ETEC challenge on disaccharidase activity; however, authors reported increases in maltase activity in early weaned-pigs, which was closely correlated with corresponding mRNA levels, suggesting a large impact of transcriptional regulation of maltase after weaning (Marion et al., 2005). Further investigation into how a severe enteric challenge and changes in feed intake impacts intestinal disaccharidases is warranted.

In conclusion, these results provide insight into how a severe F18 ETEC infection impacts growth performance, mortality, intestinal function, and immune response in weaned pigs. Weaned pigs challenged with F18 ETEC had reduced BW, ADFI, and ADG, which appears to have resulted from colonization of pathogenic *E. coli* as supported by increases in bacterial shedding and fecal scores, and reduced body temperature. While no apparent beneficial effects of DFM1 supplementation were observed, DFM2 appeared to partially attenuate the ETEC challenge by decreasing *E. coli* shedding earlier following inoculation, which resulted in improvements in intestinal barrier integrity and function on dpi 10. The ETEC challenge also resulted in impaired intestinal barrier integrity, shown by lower mRNA abundance of the tight junction proteins *OCLN* and *ZO-1* and reduced mucosal sIgA, as well as activation of an immune response, as evidenced by increases in both localized and systemic *IL-8* production. Overall, inclusion of DFM2 in nursery pig diets may be a useful tool to help alleviate PWD induced by an ETEC. The somewhat promising results of the DFM2 product indicate a need to conduct studies on this product on a larger scale and under a more moderate enteric health challenge.

## Abbreviations

ADG: average daily gain
ADFI: average daily feed intake
aEE: acid-hydrolyzed ether extract
BW: body weight
cfu: colony-forming units
CLDN1: claudin-1
CLDN3: claudin-3
CD14: cluster of differentiation 14
DFM: direct-fed microbial
dpi: days postinoculation
ETEC: enterotoxigenic Escherichia coli
G:F: gain to feed ratio
IFN-γ: interferon-γ
IL: interleukin
NC: Nonchallenged
OCLN: occludin
PBS: phosphate-buffer solution
PC: positive challenged control
PCR: polymerase chain reaction
PWD: postweaning diarrhea
RPL19: ribosomal protein-L19
SID: standardized ileal digestible
STTD: standardized total tract
TLR4: toll-like receptor 4
TNF-α: tumor necrosis factor-α
TSA: tryptic soy agar
ZO-1: zonula occludens-1
